# Effects of spatial location on distractor interference

**DOI:** 10.1167/jov.24.9.4

**Published:** 2024-09-06

**Authors:** Dirk Kerzel, Martin Constant

**Affiliations:** 1 Faculté de Psychologie et des Sciences de l'Education, Université de Genève, Genève, Switzerland

**Keywords:** biased competition, performance fields, attentional capture

## Abstract

When target and distractor stimuli are close together, they activate the same neurons and there is ambiguity as to what the neural activity represents. It has been suggested that the ambiguity is resolved by spatial competition between target and nontarget stimuli. A competitive advantage is conveyed by bottom-up biases (e.g., stimulus saliency) and top-down biases (e.g., the match to a stored representation of the target stimulus). Here, we tested the hypothesis that regions with high perceptual performance may provide a bottom-up bias, resulting in increased distractor interference. Initially, we focused on two known anisotropies. At equal distance from central fixation, perceptual performance is better along the horizontal than the vertical meridian, and in the lower than in the upper visual hemifield. Consistently, interference from distractors on the horizontal meridian was greater than interference from distractors on the vertical meridian. However, distractors in the lower hemifield interfered less than distractors in the upper visual hemifield, which is contrary to the known anisotropy. These results were obtained with targets and distractors on opposite meridians. Further, we observed greater interference from distractors on the meridians compared with distractors on the diagonals, possibly reflecting anisotropies in attentional scanning. Overall, the results are only partially consistent with the hypothesis that distractor interference is larger for distractors on regions with high perceptual performance.

## Introduction

Visual stimuli of interest are mostly surrounded by stimuli we are not interested in. Because of the large receptive fields in extrastriate cortex, target and nontarget stimuli may activate the same neurons, resulting in ambiguity as to what the neurons’ activity reflects. It has been suggested that the ambiguity is resolved by competition between target and nontarget stimuli (Desimone & Duncan, 1995; Luck, Girelli, McDermott, & Ford, 1997; Tsotsos et al., 1995). Desimone and Duncan (1995) suggested that the competition for in-depth processing is biased by bottom-up and top-down factors. For instance, stimuli that stand out from the other stimuli or stimuli that match stored features of the target have an advantage in the competition (Carlisle, Arita, Pardo, & Woodman, 2011; Duncan & Humphreys, 1989; Eimer, 2014; Huynh Cong & Kerzel, 2021; Luck, Gaspelin, Folk, Remington, & Theeuwes, 2021; Schneider, 2013). A strong prediction of the biased competition account is that the distance between stimuli should matter. With short distances between two stimuli, chances are higher that the two stimuli activate the same neurons, making it more difficult to disambiguate the target. Therefore, competition should be stronger for short compared with long distances. Support for this idea comes from search tasks where an inconspicuous target appeared in an array of nontargets. On some trials, one of the nontargets, the distractor, was salient and appeared at various distances from the target. Consistent with biased competition, interference from the distractor was found to be stronger for short compared with long distances between target and distractor (Barras & Kerzel, 2016; Caputo & Guerra, 1998; Feldmann-Wustefeld, Weinberger, & Awh, 2021; Gaspar & McDonald, 2014; Hickey & Theeuwes, 2011; Jannati, Gaspar, & McDonald, 2013; Kerzel & Huynh Cong, 2022; Mathot, Hickey, & Theeuwes, 2010; Mounts, 2000; Mounts, McCarley, & Terech, 2007).

Although distance effects have been documented amply, little is known about how spatial competition from salient distractors is affected by their location in the visual field. Perceptual performance is not equal across the visual field but varies as a function of distance and direction from the fixation point. Performance declines with increasing distance from fixation (e.g., Cannon, 1985; Rijsdijk, Kroon, & van der Wildt, 1980), but even at the same distance from central fixation, performance varies as a function of direction. In the current contribution, we focused on two known anisotropies with respect to the vertical and horizontal meridians. Previous studies found performance to be better for stimuli on the horizontal meridian than for stimuli on the vertical meridian. This anisotropy was confirmed in a variety of tasks, such as the perception of low-contrast stimuli (e.g., Carrasco, Talgar, & Cameron, 2001; Fuller, Rodriguez, & Carrasco, 2008; Hanning, Himmelberg, & Carrasco, 2022; Rovamo & Virsu, 1979), the perception of fine spatial detail (e.g., Altpeter, Mackeben, & Trauzettel-Klosinski, 2000; Greenwood, Szinte, Sayim, & Cavanagh, 2017; Talgar & Carrasco, 2002) and the processing of numerosity (Chakravarthi, Papadaki, & Krajnik, 2022). Further, performance along the vertical meridian was found to be better in the lower than the upper visual hemifield (Carrasco et al., 2001; He, Cavanagh, & Intriligator, 1996). The change in performance from the horizontal to the vertical meridian or from the lower to the upper hemifield is gradual (Abrams, Nizam, & Carrasco, 2012; Barbot, Xue, & Carrasco, 2021). The reason for the better performance is the higher density of cones and retinal ganglion cells, but cortical magnification also contributes (Kupers, Benson, Carrasco, & Winawer, 2022).

Thus, a large body of research showed that perceptual performance is better along the horizontal than the vertical meridian and in the lower than the upper hemifield. These regions of improved performance are referred to as high-performance regions. In the context of biased competition theory, the question arises whether better perceptual performance would affect the resolution of spatial competition between target and distractor stimuli. That is, would a distractor in a high-performance region interfere more with target processing than a distractor in a low-performance region? High perceptual performance may be considered a bottom-up bias in favor of the distractor because its neural representation is amplified. Thus, we expect stronger spatial competition from distractors in high-performance regions.

## Experiment

**Figure 1.**
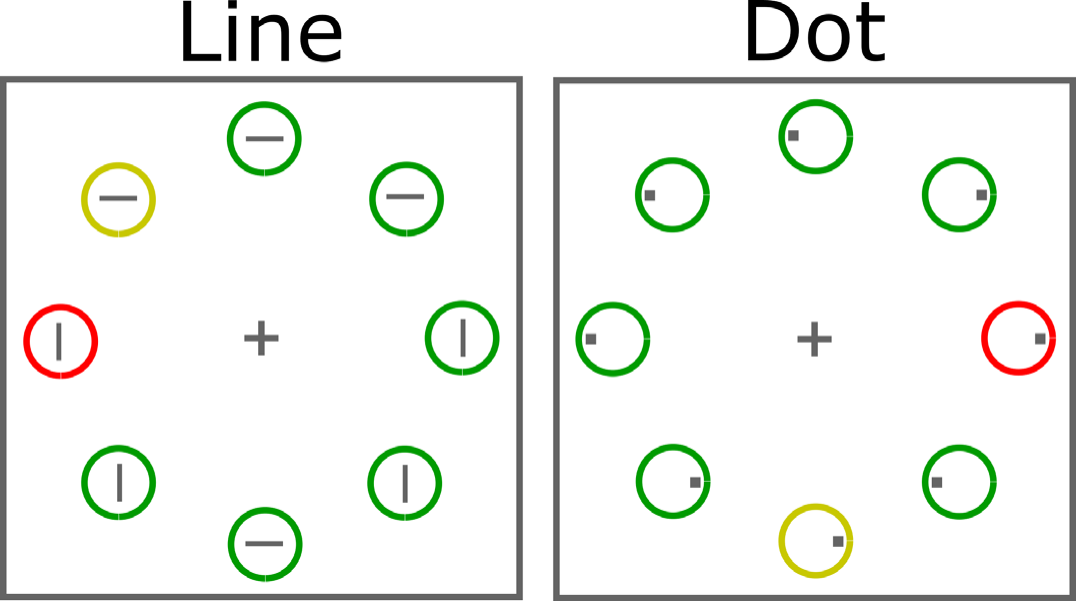
Experimental stimuli. The *yellow* target circle is shown with a *red* distractor circle among *green* nontarget circles. The line task required participants to judge the orientation of the line inside the target circle. The dot task required them to judge the location of the small square inside the target circle.

We relied on a paradigm that is known to produce robust effects of spatial competition (e.g., Gaspar & McDonald, 2014; Kerzel & Huynh Cong, 2022), but decreased the set size from ten to eight stimuli to equally sample the meridians and diagonals. Four circles were presented on the meridians and another four on the diagonals (Figure 1). On distractor-absent trials, there were seven green nontarget circles and one yellow target circle. On distractor-present trials, one of the green nontarget circles was replaced by a red distractor circle. Because the distractor was more salient than the target, strong spatial competition is expected. We presented all possible combinations of target and distractor placement in random order to avoid biases in search behavior. To test our experimental hypotheses, however, we only analyzed certain trial types.

We had two main experimental hypotheses. First, we expected more competition and therefore longer RTs for distractors on the horizontal compared with the vertical meridian. Second, we expected more competition for distractors in the lower compared with the upper visual hemifield.

Further, we changed the task between two groups of participants to evaluate stimulus-specific effects that may lead to spurious anisotropies. Both tasks were compound tasks that required participants to report a secondary feature of the target. In the line task, participants reported the orientation of a line inside the target. In the dot task, they reported the location of a dot inside the target. We do not expect the specific task to modulate the pattern of results strongly because the tasks have been used interchangeably in the literature. For instance, electrophysiological support for attentional suppression was provided using the line (Gaspar & McDonald, 2014) or the dot task (Gaspelin & Luck, 2018a) without discussion of task-specific effects.

## Methods

### Participants

We had 24 datasets for judgments of line orientation (7 men, age: *M* = 25, *SD* = 9) and 24 for judgments of dot location (8 men, age: *M* = 23, *SD* = 3). The effect size was difficult to estimate based on previous research because the relation between high-performance regions and distractor interference had not been investigated before. According to G*Power 3.1 (Faul, Erdfelder, Buchner, & Lang, 2009), we were able to detect effect sizes with a minimal *F*(1, 46) = 4.05 or η_p_^2^ = 0.081 (alpha = 0.05, power = 0.80) with 48 participants for the main effect of distractor meridian. The study was approved by the ethics committee of the Faculty of Psychology and Educational Sciences of the University of Geneva (CUREG-2022-02-23) and was carried out in accordance with the Code of Ethics of the World Medical Association (Declaration of Helsinki). Informed consent was given before each experiment.

### Apparatus and stimuli

The stimuli were displayed on a 22.5-inch LCD monitor at 100 Hz with a spatial resolution of 1,920 × 1,200 pixels (VIEWPixx Lite, standard backlight, VPixx Technologies Inc., Saint-Bruno, Canada). Colors were measured with an i1Display Pro (VPixx Edition) colorimeter by X-Rite (Grand Rapids, MI). Participants responded on a RESPONSEPixx Handheld five-button response box (VPixx Technologies Inc.). In the line task, the left key was mapped on horizontal lines and the top key on vertical lines. In the dot task, the left key was mapped on left dots and the right key on right dots. Participants pressed the keys with the index finger of their left or right hand. The Psychtoolbox (Brainard, 1997; Kleiner, Brainard, & Pelli, 2007) managed stimulus presentation and response collection.

In the illustration of the stimuli in Figure 1, sample stimuli are shown on a white background to increase visibility, but a black background was used in the experiments. In the following, stimulus dimensions are specified in degrees of visual angle. A gray fixation cross with a diameter of 0.5° and a line width of 0.04° was shown throughout in the center of the screen. The target circle was yellow, the distractor circle was red, and the remaining circles were green. The stimulus dimensions and colors were based on Gaspar and McDonald (2014), but the set size was reduced from 10 to 8 and the distances and dimensions were adjusted accordingly. We presented eight circles with a diameter of 2.7° at equal distance from each other and the fixation cross. That is, the circles were placed on the meridians and the diagonals, and the center of each circle was 7.2° away from the center of the fixation cross. For judgments of line orientation, a vertical or horizontal line was drawn inside each circle (length of 0.7°, width of 0.2°). For judgments of dot location, a small square with a side length of 0.35° was shown at 0.8° to the left or right of the center of each circle. The lines or squares inside the circles were gray. The CIE1931 *x–y* coordinates of the colors were as follows: red = (0.66, 0.31), green = (0.1, 0.72), yellow = (0.39, 0.51), and gray = (0.27, 0.35). The luminance of all stimuli was 8 cd/m^2^.

### Procedure

At the start of a trial, the fixation cross was presented for a randomly selected duration from the interval between 750 and 1,250 ms. Next, the search display was shown for 200 ms and participants searched for the yellow target circle. Participants were instructed to keep their eyes on the central fixation cross, to ignore the red circle, and to respond as quickly as possible while making < 10% errors. Immediate visual feedback informed participants about choice errors and RTs outside the response window. The response window was from 200 ms to 2,000 ms after onset of the search display. Every 84 trials, the median reaction time (RT) and error rate of the preceding 84 trials were shown during a self-determined break of at least 2 seconds. Before starting the experiment, participants completed at least 42 practice trials and were given the option to continue until they felt comfortable with the task.

### Design

The 56 possible combinations of target and distractor location and the 8 possible target locations on distractor-absent trials were combined with each of the two possible responses. Distractor-present and -absent trials made up two-thirds and one-third of the total trials, respectively. To increase the number of trials of interest, we increased the proportion of distractor-present trials to 67% compared with 50% in previous studies (Gaspar & McDonald, 2014; Kerzel & Huynh Cong, 2022). Possibly, a greater proportion of distractor-present trials decreases distractor interference (Bogaerts, van Moorselaar, & Theeuwes, 2022; Moher, Abrams, Egeth, Yantis, & Stuphorn, 2011; Won, Kosoyan, & Geng, 2019). However, we were interested mostly in comparisons among distractor-present trials and only used distractor-absent trials as baseline to control for effects of position. Participants worked through 1,344 trials, which took 47 minutes on average. Task was a between-participant variable; the remaining variables were within-participant. One-half of the participants performed the line task and the other half the dot task.

### Analyses

The data and analysis scripts in Python are available at https://osf.io/9jae4/ in the Open Science Framework. In multiple paired *t* tests, we controlled the false discovery rate (Benjamini & Hochberg, 1995), but the uncorrected *p* values are reported for clarity. These *t* tests remained significant after correction. RTs outside the response window were excluded (<0.1%). Subsequently, trials with errors were rejected and then data were trimmed for each participant and condition by removing trials with RTs that were more than 2.5 *SD*s above the respective condition's mean. This resulted in the exclusion of an additional 2% to 3% of the trials. Before running mixed analyses of variance (ANOVAs) with task as a between-participant variable, we checked the homogeneity of variances. Levene's test revealed no significant difference between the task groups in any of the conditions entering ANOVA.

## Results

**Figure 2.**
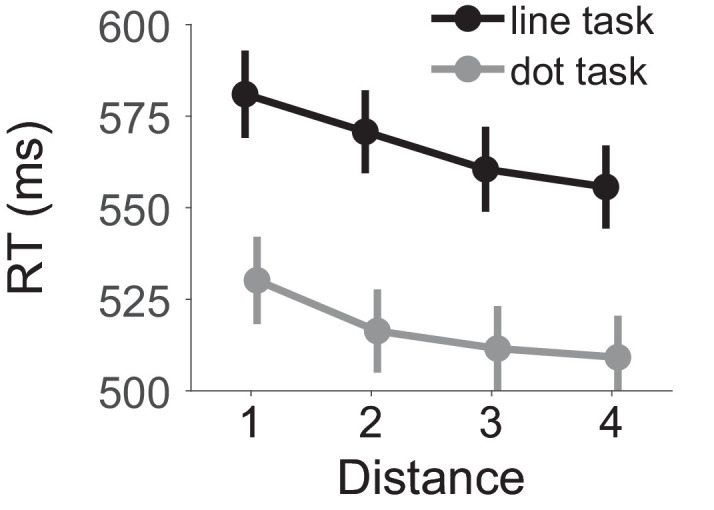
RTs as a function of task and distance between target and distractor. Error bars show the between-participant standard error of the mean.

As a manipulation check, we evaluated effects of target-distractor distance. We expected longer RTs for short distances because competition is stronger. We conducted a 2 (task: line, dot) × 4 (distance: 1, 2, 3, 4) mixed ANOVA on mean individual RTs. The full ANOVA tables are available on OSF (RT: https://osf.io/y7dh5/; Errors rates: https://osf.io/5q64e/). The group means are shown in in Figure 2. Consistent with spatial competition, RTs were longer with short distances between target and distractor, *F*(3, 138) = 82.84, *p* < 0.001, η_p_^2^ = 0.643. Mean RTs were 555, 544, 536 and 532 ms for distances of 1, 2, 3, and 4, respectively. RTs decreased by 12 ms from distance 1 to 2, *t*(47) = 9.38, *p* < 0.001, Cohen's *d_z_* = 1.35, by 8-ms from distance 2 to 3, *t*(47) = 5.89, *p* < 0.001, Cohen's *d_z_* = 0.85, and by 4-ms from distance 3 to 4, *t*(47) = 2.61, *p* = 0.012, Cohen's *d_z_* = 0.38. In addition, RTs were 50 ms longer in the line task than in the dot task (567 vs. 517 ms), *F*(1, 46) = 9.60, *p* = 0.003, η_p_^2^ = 0.173. A similar ANOVA on the percentage of choice errors confirmed the effect of distance, *F*(3, 138) = 2.74, *p* = 0.046, η_p_^2^ = 0.056. The mean percentages for each distance were 5.9%, 5.2%, 5.4%, and 5.0%. The interaction between distance and task was not significant, neither in RTs, *F*(2.13, 95.94) = 2.44, *p* = 0.089, η_p_^2^ = 0.050, nor in error percentages, *F*(3, 138) = 1.37, *p* = 0.254, η_p_^2^ = 0.029. Thus, RTs to targets with close distractors were longer than to targets with far distractors, which reflects stronger spatial competition.

### Rationale for analyses of distractor position

We had two main hypotheses. First, we expected distractors on the horizontal meridian to cause more interference than distractors on the vertical meridian. Second, we expected distractors in the lower visual field to cause more interference than distractors in the upper visual field. To answer these questions, we performed hypothesis-driven tests on selected conditions. Isolating the relevant conditions was necessary because the simultaneous analysis of the 54 target-distractor configurations with eight unique positions and four different target-distractor distances is not feasible. For these hypothesis-driven tests, the choice of positions was constrained by the distance between target and distractor, which must be controlled for because distractor interference changes with distance.

For the comparison between horizontal and vertical distractors, the easiest solution is to use diagonal targets, which are separated from the meridian distractors by a distance of one. However, as we will show below, this analysis did not yield any significant results. Thus, we focused on a target-distractor distance of two where target and distractor are placed on opposite meridians, which makes it necessary to disentangle effects of target and distractor meridian. To this end, we evaluated RTs with horizontal and vertical distractors relative to the same target positions, but without the distractor.

For the comparison between upper and lower hemifield, however, the target was always placed on the horizontal meridian. Therefore, it was not necessary to account for effects of target position.

### Target–distractor distance of two

#### Distractors on the horizontal versus vertical meridian

**Figure 3.**
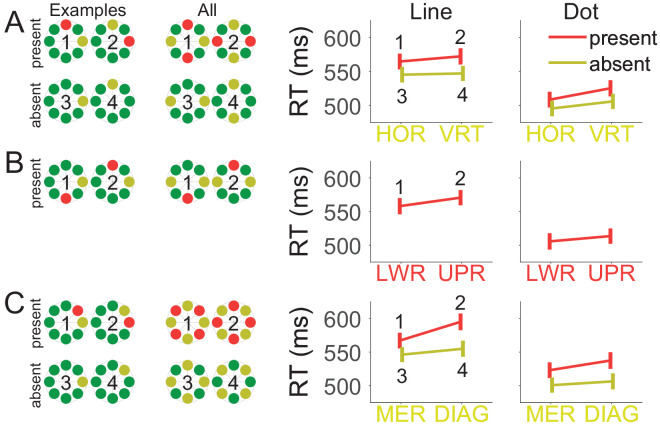
Illustration of the analyses and experimental results. Each row (**A**–**C**) shows illustrations and results for an analysis reported in the text. (**A**) Comparison between horizontal and vertical meridians. (**B**) Comparison between the upper and lower visual field. (**C**) Comparison between meridians and diagonals. Targets are represented in *yellow*, distractors in *red*, and nontargets in *green*. The first column shows example trials. The second column shows all possible target and distractor locations. The numbers in the illustrations in columns 1 and 2 match the conditions in columns 3 and 4. Note that in **A** and **C**, the *x*-axis represents the target (and not the distractor) locations. Error bars represent the between-participant standard error. DIAG, diagonal; Dot, dot task; HOR, horizontal; Line, line task; LWR, lower; MER, meridian; UPR, upper; VRT, vertical.

We examined whether distractor interference was larger for distractors on the horizontal than vertical meridian. To illustrate the comparison, we show examples of target and distractor locations in Figure 3. As shown in Figure 3A, we compared distractors on the vertical meridian with distractors on the horizontal meridian. To keep the same distance between target and distractor, we only analyzed trials where target and distractor were on opposite meridians (i.e., horizontal distractor with vertical target and the other way around). To partial out effects of target location, we included distractor-absent trials in our analysis. Therefore, we had to run the ANOVA with target location (and not distractor location) as the factor. That is, in Figure 3A, the *x*-axis shows horizontal vs. vertical target location (HOR vs. VRT). We conducted a 2 (task: line, dot) × 2 (distractor: present, absent) × 2 (target meridian: horizontal, vertical) mixed ANOVA. The full ANOVA tables are available on OSF (RT: https://osf.io/upkzg/; Errors rates: https://osf.io/sbny8/). RTs were longer on distractor-present than distractor-absent trials (543 ms vs. 523 ms), *F*(1, 46) = 89.59, *p* < 0.001, η_p_^2^ = 0.661. Importantly, the effect of distractor presence was modulated by target meridian, *F*(1, 46) = 5.06, *p* = 0.029, η_p_^2^ = 0.099. The difference between distractor-present and distractor-absent trials was 17 ms when the distractor was shown on the vertical meridian (with HOR targets), but this difference was greater (22 ms) when the distractor was shown on the horizontal meridian (with VRT targets). Thus, we find that presenting distractors on the horizontal meridian results in stronger interference than presenting them on the vertical meridian, at least with targets on the opposite meridian. This result is consistent with the hypothesis that high-performance regions provide a bottom-up bias in the competition between target and distractor.

Further, there were some unpredicted results that do not directly speak to our hypotheses. RTs were longer in the line than in the dot task (557 ms vs. 509 ms), *F*(1, 46) = 9.24, *p* = 0.004, η_p_^2^ = 0.167. The effect of target meridian, *F*(1, 46) = 22.48, *p* < 0.001, η_p_^2^ = 0.328, was modulated by task, *F*(1, 46) = 5.28, *p* = 0.026, η_p_^2^ = 0.103. In the line task, RTs were about the same for targets on the horizontal and vertical meridian (555 ms vs. 560 ms), *t*(23) = 1.73, *p* = 0.091, Cohen's *d_z_* = 0.353, whereas in the dot task, RTs were shorter for targets on the horizontal than vertical meridian (502 ms vs. 515 ms), *t*(23) = 4.98, *p* < 0.001, *d_z_* = 1.02. Further, the analysis of choice errors confirmed worse performance in the presence than absence of a distractor (5.4% vs. 3.8%), *F*(1, 46) = 40.47, *p* < 0.001, η_p_^2^ = 0.468. In addition, there were more errors for targets on the horizontal than vertical meridian (5.2% vs. 4.1%), *F*(1, 46) = 7.87, *p* = 0.007, η_p_^2^ = 0.146.

#### Distractors in the lower versus upper hemifield

We evaluated whether distractor interference was greater in the lower than in the upper hemifield. To this end, we examined trials where the target was on the horizontal meridian and the distractor was on the vertical meridian either in the lower (LWR) or upper (UPR) visual hemifield (see Figure 3B). We conducted a 2 (task: line, dot) × 2 (distractor hemifield: lower, upper) mixed ANOVA. The full ANOVA tables are available on OSF (RT: https://osf.io/npzka/; Errors rates: https://osf.io/8ry6j/). We found that RTs were shorter with meridian distractors located in the lower than in the upper visual hemifield (532 ms vs. 542 ms), *F*(1, 46) = 9.16, *p* = 0.004, η_p_^2^ = 0.166, suggesting that meridian distractors in the lower hemifield interfered less than distractors in the upper hemifield. We also replicated longer RTs in the line than in the dot task (565 ms vs. 509 ms), *F*(1, 46) = 11.60, *p* = 0.001, η_p_^2^ = 0.201. Running the same ANOVA on the percentage of choice errors did not find any significant effects (*p*s > 0.114).

Thus, there was less interference from distractors on the vertical meridian in the lower than upper hemifield, which is inconsistent with the presumed boost of distractors in the lower visual hemifield. Although the results refute our initial hypothesis, several post hoc explanations apply. For instance, it could be that reduced distractor interference in the lower visual field was an expression of performance improvement. For instance, it may be that distractor suppression (Dent, Allen, Braithwaite, & Humphreys, 2012; Gaspelin & Luck, 2018b; Geng, 2014) was improved in the lower visual field, resulting in less interference from distractors in the lower than the upper visual hemifield. However, this explanation is post hoc and requires further experiments. Further, we only investigated distractor positions on the vertical meridian, and it may be that a more comprehensive test would reveal the predicted differences.

### Target–distractor distance of 1

#### Diagonal targets with vertical or horizontal distractors

These results suggest that there are differences between the horizontal and vertical meridian and between the lower and upper visual hemifield. These results were obtained with a target–distractor distance of two. As mentioned in the section on the rationale for our analysis, we explored whether similar results could be observed with a target–distractor distance of one. To this end, we analyzed trials where the target was on a diagonal location and the distractor was adjacent, either on the horizontal or vertical meridian. However, the 2 (task: line, dot) × 2 (distractor meridian: horizontal, vertical) ANOVA found no main effect of distractor meridian, *F*(1, 46) = 0.09, *p* = 0.771, η_p_^2^ = 0.002, nor an interaction with task *F*(1, 46) = 2.15, *p* = 0.150, η_p_^2^ = 0.045. The full ANOVA tables are available on OSF (RT: https://osf.io/fs23e/; Errors rates: https://osf.io/m8nwy/).

#### Diagonal targets with meridian distractors or vice versa

Further exploration of the data revealed, however, that this anisotropy may have been overshadowed by a larger anisotropy between diagonal and meridian locations. The following analysis shows that with a target–distractor distance of one (see Figure 3C), interference was larger with meridian than diagonal distractors. To partial out effects of target location, we included distractor-absent trials in our analysis and ran the ANOVA with target location (instead of distractor location) as factor. Thus, in Figure 3C, the x-axis represents target locations (MER vs. DIAG) and the relevant distractor location is opposite (i.e., diagonal for MER targets and meridian for DIAG targets).

We conducted a 2 (task: line, dot) × 2 (distractor: present, absent) × 2 (target location: meridian, diagonal) mixed ANOVA. The full ANOVA tables are available on OSF (RT: https://osf.io/cnhdx/; Errors rates: https://osf.io/hzk64/). The stronger interference from meridian than diagonal distractors was visible in a two-way interaction of target location with distractor presence, *F*(1, 46) = 67.43, *p* < 0.001, η_p_^2^ = 0.595. For meridian targets (MER), RTs increased by 22 ms with diagonal distractors, but for diagonal targets (DIAG), RTs increased by 36 ms with meridian distractors. In other words, there was more interference from distractors on a meridian (with DIAG targets) than from distractors on a diagonal (with MER targets). The three-way interaction with task was also significant, *F*(1, 46) = 4.77, *p* = 0.034, η_p_^2^ = 0.094, showing that the difference between diagonal and meridian distractors was larger in the line task (21 ms vs. 40 ms) than in the dot task (23 ms vs. 31 ms). However, separate ANOVAs showed that the two-way interaction between target location and distractor presence was significant in both the dot task, *F*(1, 23) = 8.64, *p* = 0.007, η_p_^2^ = 0.273, and the line task, *F*(1, 23) = 21.25, *p* < 0.001, η_p_^2^ = 0.480.

Further, there were some results that either replicate previous analyses or are not directly relevant for our hypotheses. As shown elsewhere in this article, RTs were longer in the line than in the dot task (566 ms vs. 517 ms), *F*(1, 46) = 8.88, *p* = 0.005, η_p_^2^ = 0.162, and on distractor-present than -absent trials (556 ms vs. 527 ms), *F*(1, 46) = 213.92, *p* < 0.001, η_p_^2^ = 0.823. Further, RTs were overall shorter with meridian than diagonal targets (534 ms vs. 549 ms), *F*(1, 46) = 67.43, *p* < 0.001, η_p_^2^ = 0.595, and this effect was stronger in the line than in the dot task (difference of 18 ms vs. 10 ms), *F*(1, 46) = 6.17, *p* = 0.017, η_p_^2^ = 0.118.

The analysis of choice errors confirmed the results observed in RTs. Importantly, the interaction of target location and distractor presence, *F*(1, 46) = 11.43, *p* = 0.001, η_p_^2^ = 0.199, showed that distractor interference was stronger for diagonal targets (4.1% vs. 7.0%) than for meridian targets (3.8% vs. 4.8%). Thus, distractors on a meridian caused more interference than distractors on a diagonal. Further, more errors occurred on distractor-present than distractor-absent trials (5.9% vs. 3.9%), *F*(1, 46) = 36.15, *p* < 0.001, η_p_^2^ = 0.440, and with diagonal than meridian targets (5.6% vs. 4.3%), *F*(1, 46) = 11.23, *p* = 0.002, η_p_^2^ = 0.196.

## Discussion

We investigated whether regions with better perceptual performance resulted in increased competition in a visual search task. We initially focused on two known anisotropies. Performance is known to be better (1) on the horizontal than the vertical meridian and (2) in the lower than the upper hemifield (e.g., Abrams et al., 2012; Altpeter et al., 2000; Barbot et al., 2021; Carrasco et al., 2001; Chakravarthi et al., 2022; Fuller et al., 2008; Greenwood et al., 2017; Hanning et al., 2022; Rovamo & Virsu, 1979; Talgar & Carrasco, 2002). We reasoned that distractors in these high-performance regions would elicit more interference than distractors in lower-performance regions. That is, more competition was expected from distractors on the horizontal than the vertical meridian and from distractors in the lower than in the upper visual hemifield. To test this prediction, we used a visual search task generating strong spatial competition (e.g., Gaspar & McDonald, 2014; Kerzel & Huynh Cong, 2022) in which participants searched for a color target and responded based on a feature inside the target. On most trials, a more salient color distractor was shown.

RTs were longest when the distance between target and distractor was short, indicating that spatial competition occurred. We also found that more interference occurred with distractors on the horizontal than the vertical meridian, which is consistent with increased competition from distractors in high-performance regions. However, less interference occurred with distractors in the lower than the upper hemifield, which is inconsistent with our hypothesis. Note that these results were obtained with target and distractor on opposite meridians and a target–distractor distance of two.

Thus, we found some evidence for the hypothesis that there is a competitive advantage for stimuli in high-performance regions, suggesting that not only characteristics of the stimulus provide bottom-up biases (e.g., Bruce & Tsotsos, 2009; Itti & Koch, 2001; Liesefeld, Liesefeld, Pollmann, & Müller, 2019), but that its location does, too. These effects of location are consistent with “regional variations” of bottom-up stimulus activations mentioned in Guided Search 2.0 (Wolfe, 1994). However, there may also be differences between the current visual search task and the previous literature on anisotropies in low-level visual perception. Arguably, visual search is more strongly influenced by top-down factors (Carlisle et al., 2011; Duncan & Humphreys, 1989; Eimer, 2014; Huynh Cong & Kerzel, 2021; Luck, Gaspelin, Folk, Remington, & Theeuwes, 2021; Schneider, 2013; Wolfe, 1994; Wolfe, 2021). For instance, we observed stronger interference from distractors on a meridian than from distractors on a diagonal. So far, this anisotropy between meridians and diagonals has not been reported in the literature on regional variations in visual performance.

Perhaps the increased interference for stimuli on the meridians is caused by spatially systematic scanning (e.g., Liesefeld & Müller, 2020). For instance, participants may have scanned stimuli on the meridians before stimuli on the diagonals. Such systematic scanning strategies have been reported in the literature on an electrophysiological index of attentional deployment, the N2pc-component (Drisdelle & Eimer, 2021; Kerzel & Burra, 2020; Woodman & Luck, 1999). If there was a top-down bias to attend to stimuli on the meridians before stimuli on the diagonals, distractor interference would be larger for meridian than diagonal distractors. In a similar vein, top-down biases may explain why distractor interference was decreased in the lower visual hemifield. Notably, attentional suppression is thought to arise in situations where top-down control is strong (Gaspelin, Leonard, & Luck, 2015) and top-down control may be stronger in the lower visual hemifield, possibly because attentional resolution is better (He et al., 1996). The experimental conditions may have favored top-down control because of the relatively large proportion of distractor-present trials in the current study (Bogaerts et al., 2022; Moher et al., 2011; Won et al., 2019). Thus, the reasons for the anisotropies may not be limited to bottom-up factors such as high-performance regions but may include top-down factors such as scanning strategies or attentional suppression. To consolidate these ideas, however, more research is required. It would be good if the neural mechanisms of the anisotropy were investigated with established electrophysiological correlates of scanning and suppression, such as the P_D_-component (Gaspelin et al., 2023).

In sum, we tested whether stimuli in regions of high perceptual performance have a competitive advantage in a search task involving a salient distractor and a less salient target. Our results with targets and distractors on opposite meridians provide mixed evidence for this conjecture. Consistent with the anisotropy between horizontal and vertical meridians, we found stronger interference from distractors on the horizontal than the vertical meridian. However, distractors in the lower hemifield produced less interference than distractors in the upper visual hemifield, which is inconsistent with our hypotheses. Possibly, the decreased interference in the lower visual field may result from improved distractor suppression, but more research is required to evaluate this idea.
